# Supplementary material for: Selective cross‐linking of coinciding protein assemblies by in‐gel cross‐linking mass spectrometry
Source: EMBO J. 2021 Jan 18;40(4):e106174. doi: 10.15252/embj.2020106174 (PMC7883291; doi:10.15252/embj.2020106174)
Supplement: Supplementary file 1 — Appendix [file EMBJ-40-e106174-s001.pdf]

Appendix for Publication

Selective cross-linking of coinciding protein assemblies by in-gel cross-linking mass-spectrometry

Johannes F. Hevler[1,2#], Marie V. Lukassen[1,2#], Alfredo Cabrera-Orefice[3], Susanne Arnold[3], Matti F. Pronker[1,2], Vojtech Franc[1,2] and Albert J.R. Heck[1,2]*

[1] Biomolecular Mass Spectrometry and Proteomics, Bijvoet Center for Biomolecular Research and Utrecht Institute for Pharmaceutical Sciences, University of Utrecht, Padualaan 8, 3584 CH Utrecht, The Netherlands

[2] Netherlands Proteomics Center, Padualaan 8, 3584 CH Utrecht, The Netherlands

[3] Radboud Institute for Molecular Life Sciences, Radboud University Medical Center, 6525 GA Nijmegen, The Netherlands

*Correspondence: Albert Heck, a.j.r.heck@uu.nl; phone: +31302536797; Address: Utrecht University, Padualaan 8, 3584 CH Utrecht, The Netherlands

# contributed equally

Appendix Table of Contents

Figure S1– Verification of in-gel cross-linking (IGX) by SDS-PAGE

Figure S2– In-gel cross-linking is highly reproducible and only marginally dependent on the concentration of the reagent

Figure S3 – Cross-linker optimization and peptide length distributions for IGX-MS and in-solution XL-MS.

Figure S4 – IGX-MS of GroEL bound to its unfolded substrate gp23.

Figure S5 – IGX-MS of C5, C6, and C5b6.

Figure S6 – Comparison of the C6 X-ray structure and IGX-MS driven structural model, whereby the C5b-binding region becomes docked to the main body.

Figure S7– Possible interactions between adjacent C6 molecules in the deposited X-ray crystal structure.

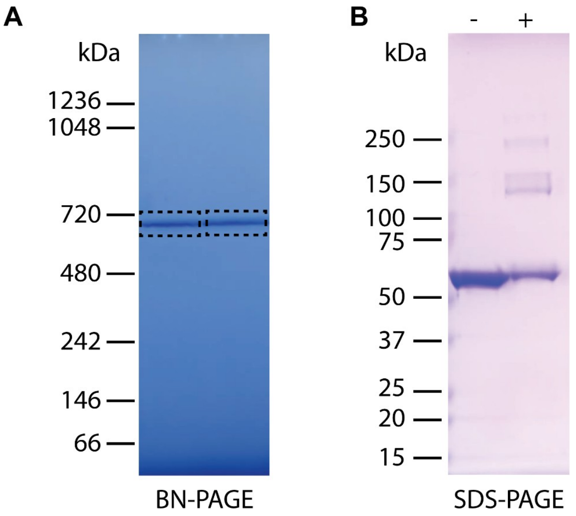

Figure S1 – Verification of in-gel cross-linking (IGX) by SDS-PAGE

A. BN-PAGE of *E. Coli* (10 µg). Respective bands (dashed boxes) were excised and incubated with or without the cross-linker DSS (1.5 mM).
B. SDS-PAGE of GroEL, extracted from respective gel band (see A). The non-cross-linked control (-) showed only a band at 57 kDa of the GroEL subunit, whereas the DSS-cross-linked sample (+) reveals several bands at higher Mw.

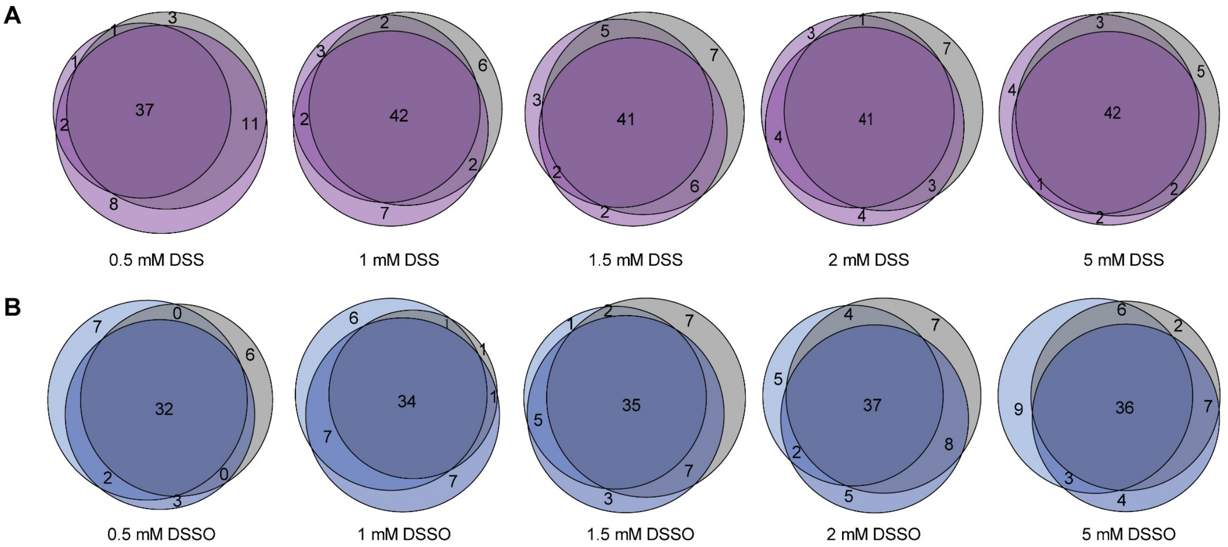

Figure S2 – In-gel cross-linking is highly reproducible and only marginally dependent on the concentration of the reagent.

A. Venn diagrams displaying the overlap of detected unique cross-links in triplicate measurements of GroEL cross-linked in gel with different DSS concentrations

B. Venn diagrams displaying the overlap of detected unique cross-links in triplicate measurements of GroEL cross-linked in gel with different DSSO concentrations.

Data information: Source data is available online (PRIDE database: PXD020014).

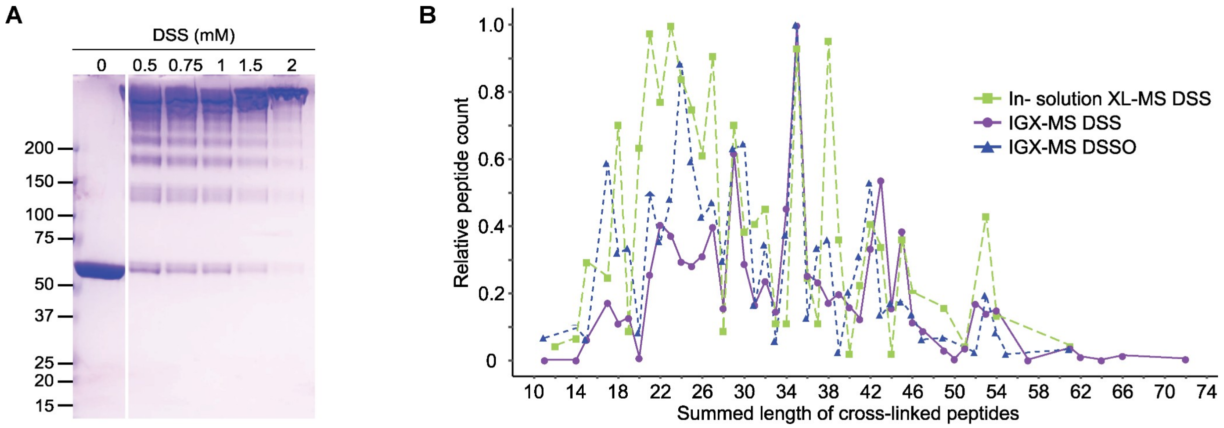

Figure S3 - Optimization of cross-linker concentration for in-solution XL-MS and observed cross-linked peptide length distributions for IGX-MS and in-solution XL-MS.

A. GroEL was cross-linked in-solution using different DSS concentrations for the optimization of the cross-linker concentration. The samples were then loaded onto a SDS-PAGE for visualization. A concentration of 0.75 mM DSS was selected for further experiments.

B. Comparison of summed length of cross-linked peptide-pairs from IGX-MS (1.5 mM DSS or 2 mM DSSO) or in-solution XL-MS (0.75 mM DSS) experiments.

Data information: Source data for identified peptides is available online (PRIDE database: PXD020014).

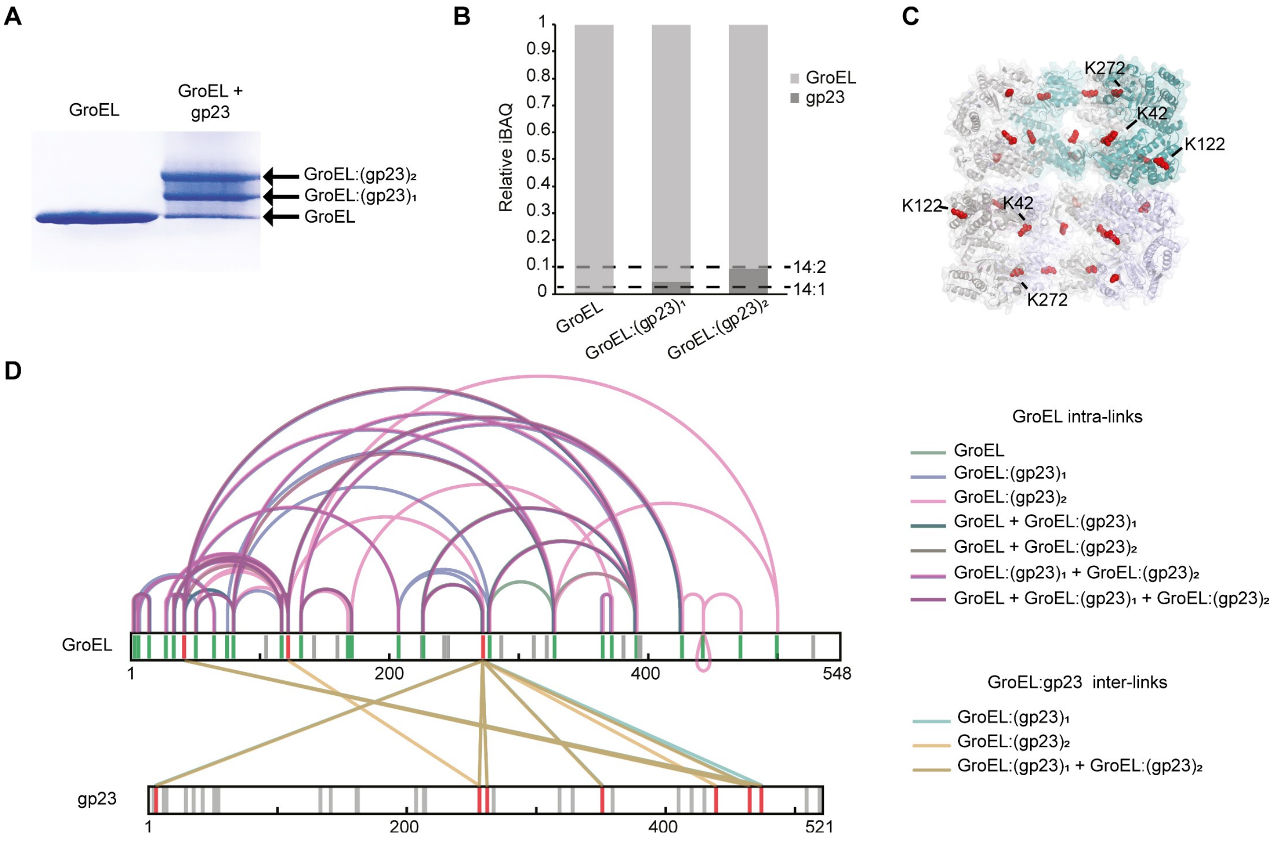

Figure S4 - IGX-MS of GroEL bound to the gp23 substrate.

A. BN-PAGE of GroEL incubated with or without unfolded gp23. The arrows indicate free GroEL or GroEL bound to one or two molecules of gp23.

B. Label-free quantification for the estimation of the stoichiometry of the formed complexes. Relative iBAQ values of GroEL and gp23 in the three bands. Dashed lines indicate the GroEL:gp23 ratios for the theoretically expected 14:1 and 14:2 ratios.

C. Cross-section of the structural model of GroEL (PDB ID: 1KP8) with lysine residues that were found to be cross-linked to gp23 shown in red spheres.

D. Overlay of cross-links identified in the three bands, representing the GroEL, the GroEL:gp23, and GroEL:$(gp23)_2$ complex. GroEL intra-links are colored green, purple, and pink. Inter-links are colored turquoise and sand. For clarity, intra-links in gp23 are not depicted. Grey lines in the sequence indicate lysine residues not cross-linked, red lines indicate inter-linked lysine residues, and green lines indicate intra-linked residues.

Data information: Source data is available in Dataset EV2. All cross-links were included in the analysis.

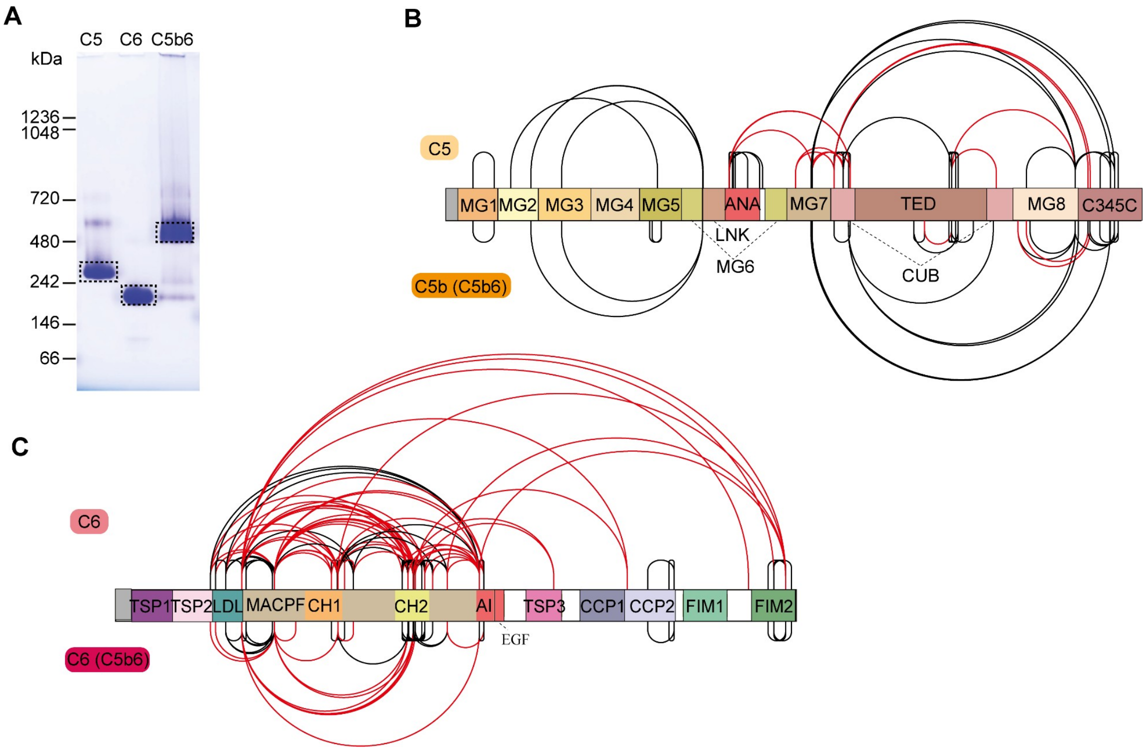

Figure S5 – IGX-MS of C5, C6, and C5b6.

A. BN-PAGE of complement components C5, C6, and the C5b6 complex. For each lane 5 µg of protein (C5, C6), respectively 10 µg (C5b6) of protein, were applied onto the gel. Dashed boxes indicate the bands cut out for subsequent IGX-MS analysis.

B, C. Schematic overview for domain-centered cross-link results for C5 and Cb5b in the C5b6 complex (B) or C6 and C6 in the C5b6 complex (C). Black lines indicate cross-links within the distance restraints (≤ 30 Å). Red lines indicate cross-links exceeding the distance restraints (≥ 30 Å). Notably, for C6, many cross-links to the N-terminal domains are not present when C6 is complexed to C5b.

Data information: Source data available in Dataset EV4. Only cross-links identified in at least two of three replicates were included in the analysis.

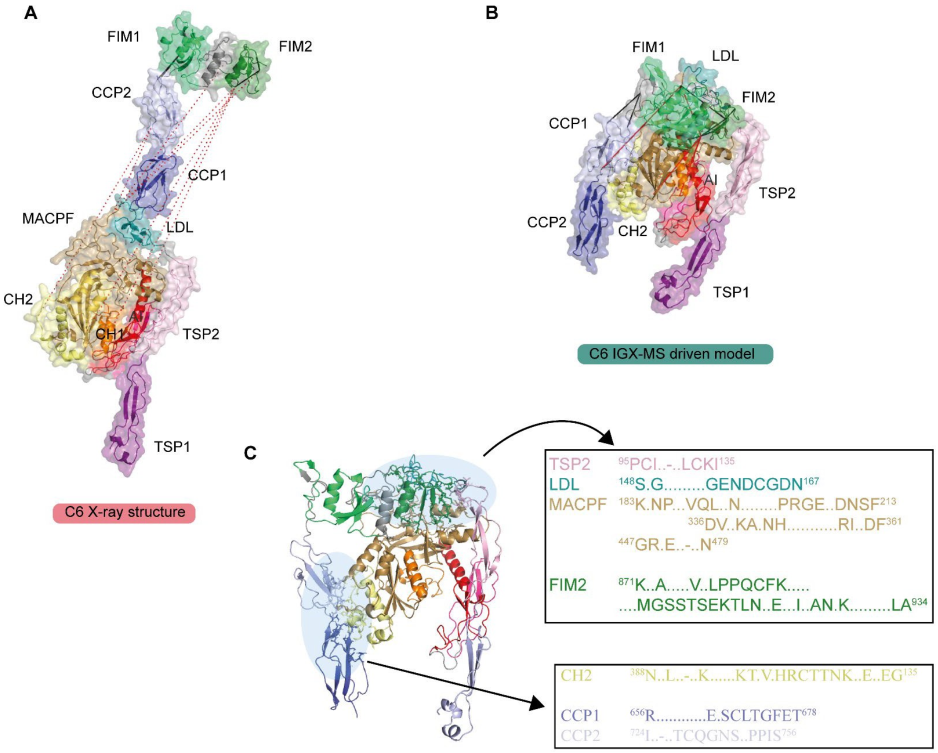

Figure S6 – Comparison of the monomeric C6 X-ray structure and IGX-MS driven structural model, wherein the C5b-binding region becomes docked to the main body.

A. Cross-links identified for residues in the C5b-binding region plotted onto the C6 X-ray structure (PDB ID: 3T5O). Cross-links that were used to dock the C5b-binding domain (CCP1-2, FIM1-2) to the LDL- and MACPF domain are shown as dotted lines. Black lines indicate cross-links within the distance restraints (≤ 30 Å). Red lines indicate cross-links exceeding the distance restraints (≥ 30 Å).

B. IGX-MS driven structural model of free C6. Cross-links obtained for the C5b-binding region are plotted onto the final model of C6. Solid black lines indicate distances below 30 Å, and red dotted lines indicate links with a distance larger than 30 Å.

C. Interaction interface analysis for C5b-binding and the LDL-, MACPF domains. Blue circles indicate binding interfaces, for which interacting residues were identified (shown as sticks). The identified interacting residues of the different domains are shown in the black boxes.

Data information: A-B: Structural coordinates were obtained from indicated models. C: Source data is available in Dataset EV5.

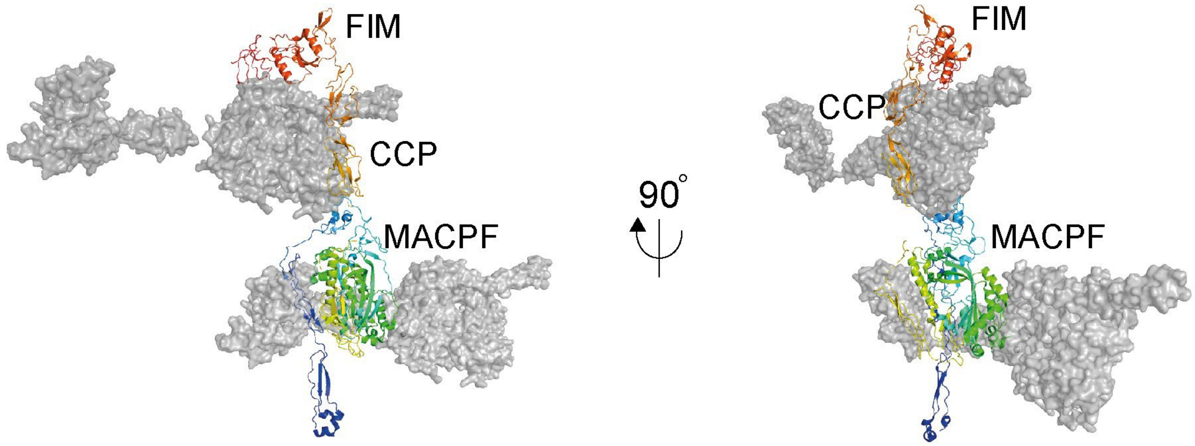

Figure S7 – Possible interactions between adjacent C6 molecules in the deposited X-ray crystal structure.

The total electron density acquired for the X-ray structure of C6 (PDB ID: 3T5O) reveals closely packed neighboring C6 molecules. A single C6 molecule is shown here in a rainbow-colored cartoon in two orientations. The surface (grey) of two adjacent C6 molecules in the electron density maps shows the interaction of the C-terminal CCP and FIM domains of one C6 molecule with the MACPF domain of another C6 molecule.

Data information: Data was obtained from the PDB database using the indicated identifier.
