# Supplementary material for: Selective cross‐linking of coinciding protein assemblies by in‐gel cross‐linking mass spectrometry
Source: EMBO J. 2021 Jan 18;40(4):e106174. doi: 10.15252/embj.2020106174 (PMC7883291; doi:10.15252/embj.2020106174)
Supplement: Supplementary file 2 — Expanded View Figures PDF [file EMBJ-40-e106174-s002.pdf]

Expanded View Figures

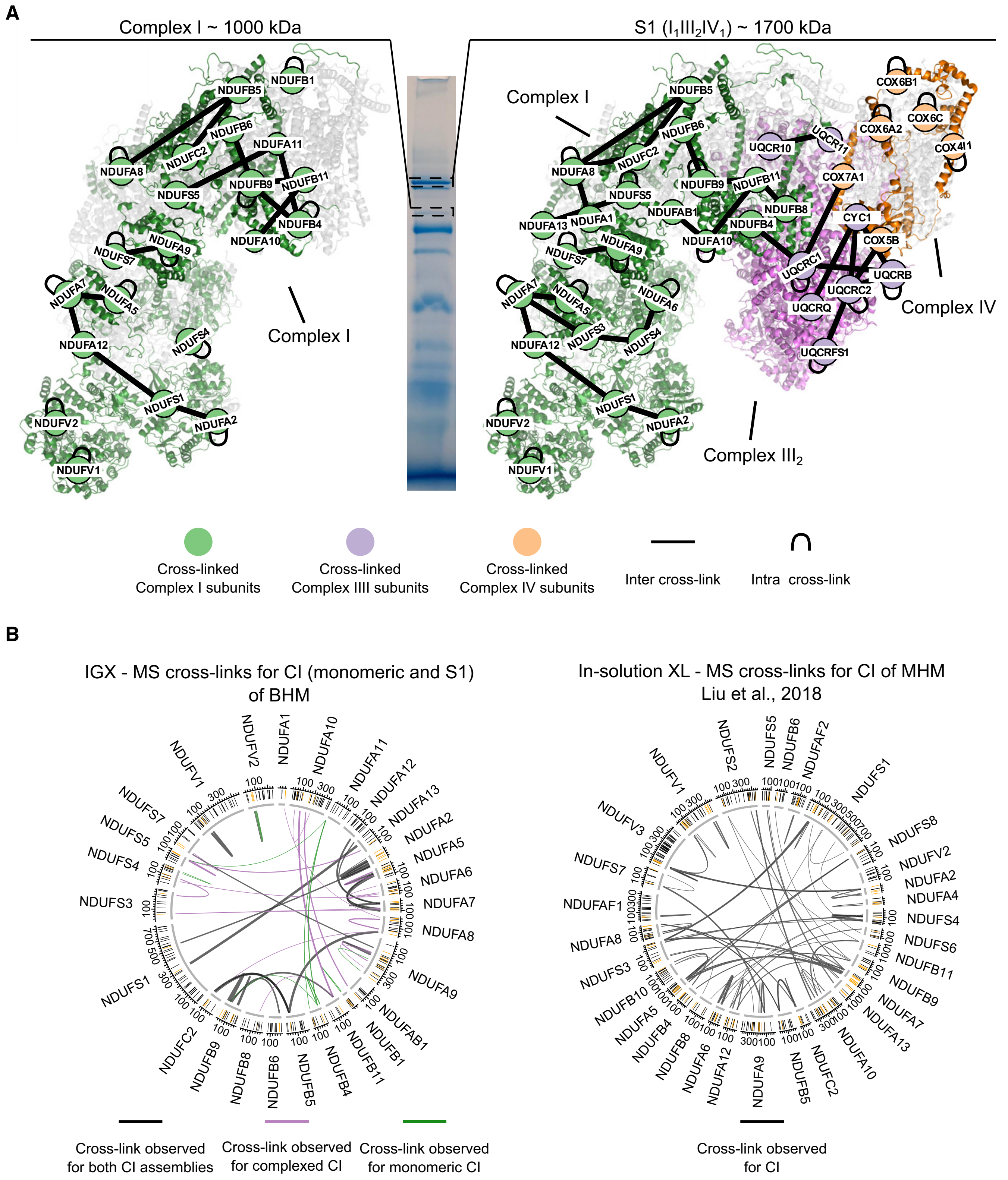

Figure 1.

Figure EV1. Assembly state-specific cross-linking of complex I from bovine heart mitochondria (BHM) in its monomeric state and when incorporated within a supercomplex by IGX-MS.

A IGX-MS cross-links observed within monomeric complex I (left)—and when incorporated within the supercomplex S1 (right). Node positions are in accordance with respective subunit coordinates of the published S1 structure (PDB ID: 5GUP).

B Circos plots of detected cross-links within the monomeric complex I (CI) (black Gene names) identified by IGX-MS (left panel) and in-solution XL-MS (right panel). The position of the lysine residues is shown in the outer-ring, and cross-linked residues are colored dark orange. For IGX-MS generated data (left panel), cross-links are colored based on the CI assembly state they were detected for. For in-solution XL-MS (right panel), the cross-links originated from a mixture of all assembly states of CI present in-solution. Thickness of the cross-link lines correlates to the number of detected cross-linked spectra matches (CSMs).

Data information: The presented IGX-MS data are summed from triplicates. For illustration, the same BN-PAGE of solubilized BHM is repeatedly shown (see Fig 4A).

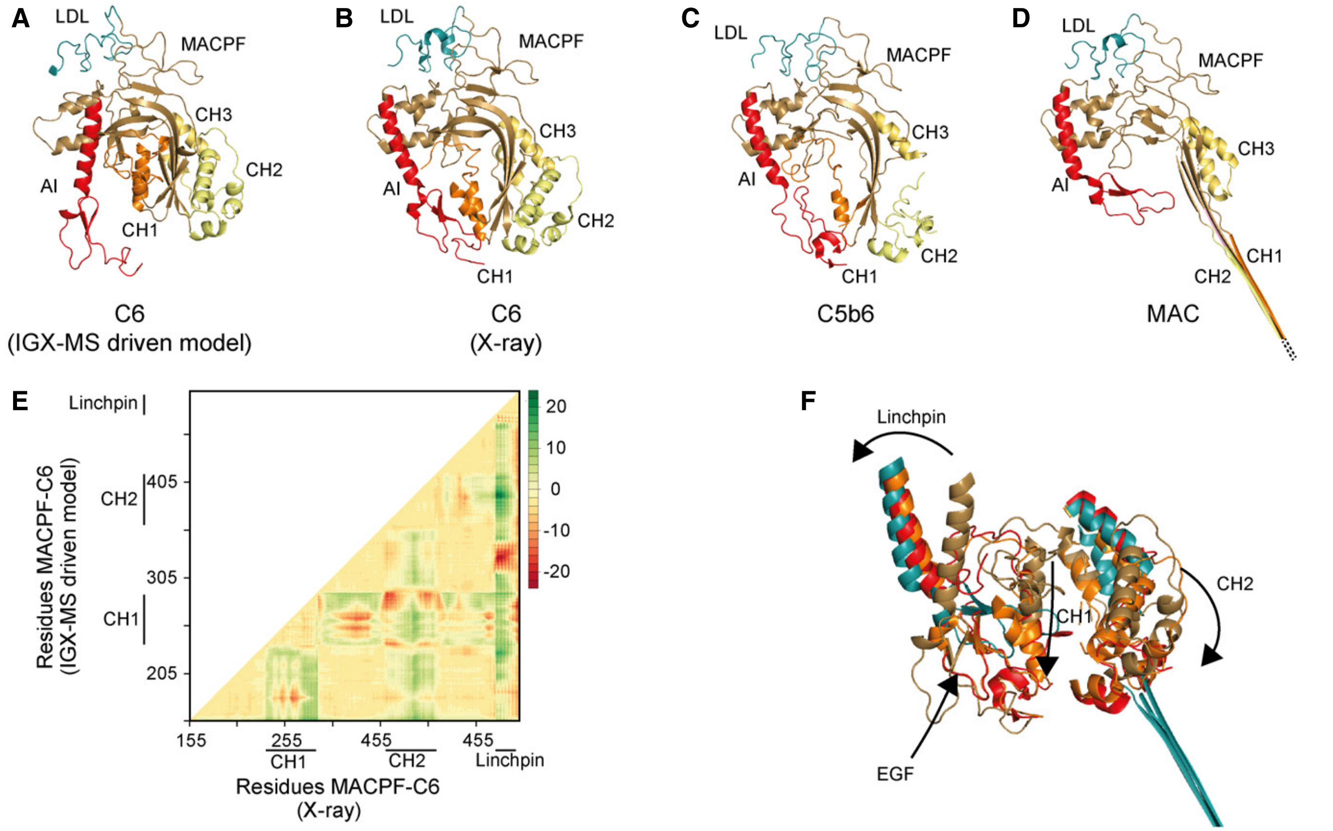

Figure EV2. Structural rearrangements of the AI and CH regions of C6 when going from free C6, the C5b6 intermediate structure to C6 in the fully assembled MAC.

A–D Cartoon representation of the LDL (deep cyan) and MACPF (sand) domains within the IGX-MS-driven C6 model (A), C6 from the X-ray structure (PDB ID: 3T5O) (B), C6 complexed with C5b (PDB ID: 4A5W) (C), and C6 as part of the MAC (PDB ID: 6H03) (D). The regions CH 1-3 (orange, pale yellow, and yellow orange) and autoinhibitory (AI, red) within MACPF are shown.

E The difference distance matrix of superposed C6-MACPF domains. The difference distance was calculated by subtracting the coordinates of aligned MACPF backbones of the IGX-MS-driven model and the I-tasser model (for complete sequence coverage) of C6-X-ray structure (PDB ID: 3T5O). The distance values are plotted with colors representing Cα differences of −20 to 20 Å according to the right-sided scale.

F Overlay of the AI (composed of linchpin helix and EGF domain), CH1, and CH2 regions of MACPF in four distinct conformations of C6, namely the IGX-MS-driven model (sand), the deposited C6 X-ray structure (orange), when incorporated in the C5b6 complex (red), and finally when incorporated in the fully assembled MAC (deep cyan). Arrows indicate the conformational changes of the different regions from free C6 (IGX-MS-driven model) to partial activation (PDB ID: 3T5O) and C5b binding (PDB ID: 4A5W) and final assembly into the MAC (PDB ID: 6H03).

Data information: MACPF structural coordinates were obtained from indicated structures and the IGX-MS-driven model.

 © 2021 The Authors

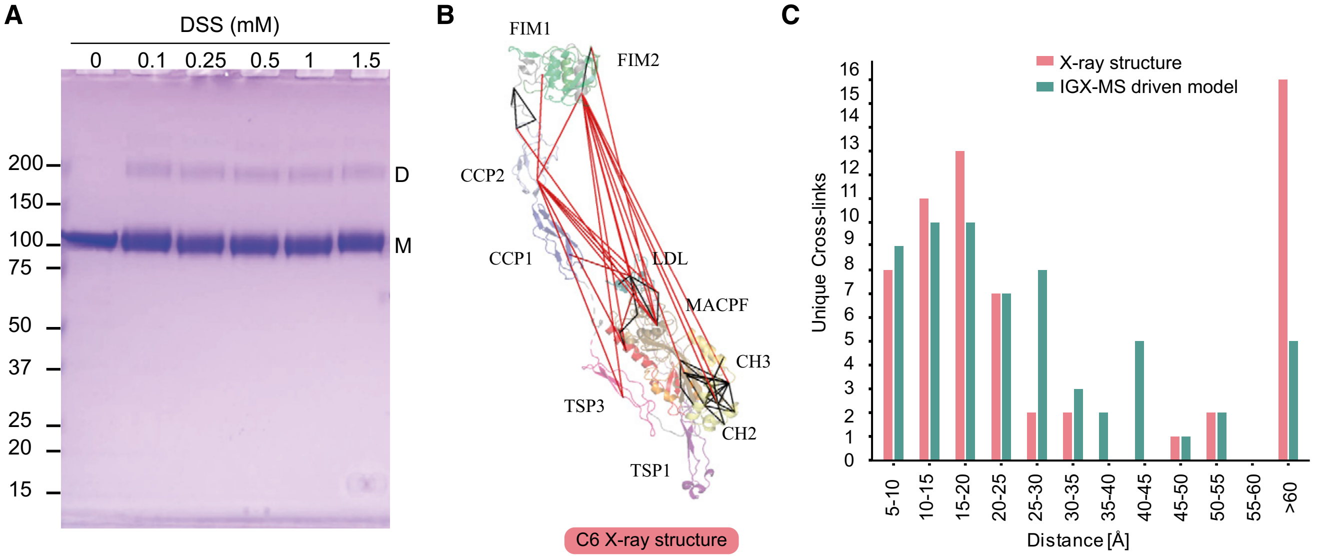

Figure EV3. In-solution XL-MS of C6.

A DSS concentration optimization for cross-linking C6 in-solution monitored by SDS–PAGE. The upper band (~200 kDa) indicates a C6 dimer that is formed upon cross-linking, whereas the more abundant lower band (~100 kDa) represents monomeric C6.

B Obtained cross-links for monomeric C6 plotted on the available X-ray structure of C6 (PDB ID: 3T5O). The red lines indicate distances > 30 Å. The different domains of C6 are indicated in black.

C Distribution of lysine Cα-Cα distances of unique cross-links identified by in-solution XL-MS for monomeric C6 when plotted on the reported X-ray structure (pink bars, PDB ID: 3T5O) and the IGX-MS-driven refined structural model (green bars). The average distance of all cross-links is 41.7 Å when using the X-ray structure which reduces to 26.2 Å for the IGX-MS-driven structural model.

Data information: Only cross-links identified in at least two of three replicates were included in the analysis.

© 2021 The Authors
